# Usability and Acceptability of a Pregnancy App for Substance Use Screening and Education: A Mixed Methods Exploratory Pilot Study

**DOI:** 10.2196/60038

**Published:** 2025-02-13

**Authors:** Haley Fitzgerald, Madison Frank, Katelyn Kasula, Elizabeth E Krans, Tamar Krishnamurti

**Affiliations:** 1 University of Pittsburgh School of Medicine, Pittsburgh, PA, United States; 2 Magee-Womens Research Institute, Pittsburgh, PA, United States; 3 Department of Obstetrics, Gynecology, and Reproductive Sciences, University of Pittsburgh School of Medicine, Pittsburgh, PA, United States; 4 Division of General Internal Medicine, University of Pittsburgh School of Medicine, 230 McKee Place, Suite 600, Pittsburgh, PA, 15213, United States, 1 412-692-4855

**Keywords:** substance use disorder, substance use screening, mHealth, mobile health apps, pregnancy, technology

## Abstract

**Background:**

Increasing opioid and other substance use has led to a crisis of epidemic proportions, with substance use now recognized as a leading cause of maternal morbidity and mortality in the United States. Interventions will only be effective if those who would benefit are identified early and connected to care. Apps are a ubiquitous source of pregnancy information, but their utility as a platform for evaluating substance use during pregnancy is unknown.

**Objective:**

This study aims to explore the usability and acceptability of a pregnancy app for opioid and other substance use screening and education.

**Methods:**

This mixed methods, exploratory pilot study examined adult pregnant people with a history of substance use who were recruited from outpatient and inpatient settings at a tertiary care obstetric hospital. After completing a baseline survey collecting demographics, substance use, and technology use, participants accessed an existing pregnancy support app for 4 weeks. Qualitative methods were used to measure the acceptability of embedding substance use screening, education, and information within the tool. App use frequency and access to substance use educational content and treatment referral information were evaluated.

**Results:**

The 28 female participants had a mean (SD) age of 31 (0.46) years; most were White (21/28, 75%) and Medicaid insured (26/28, 93%), with an annual household income of <US $30,000 (16/28, 57%). The mean gestational age at enrollment was 22 weeks. Almost half (13/28, 46%) were taking medication for opioid use disorder (methadone or buprenorphine). Other substances used included tobacco (22/28, 79%), marijuana (20/28, 71%), illicit opioids (9/28, 32%), alcohol (6/28, 21%), and stimulants (4/28, 14%), including cocaine, amphetamines, and benzodiazepines (2/28, 7%). Most (19/28, 68%) reported previously using one or more prenatal apps and 11% (3/28) cited prenatal apps as their most frequently used source of pregnancy information. After approximately 4 weeks of app exposure, 71% (20/28) logged in at least weekly, 89% (25/28) were satisfied with the app, and 96% (27/28) reported that the app was a helpful source of support. In cognitive interviews, participants reported that app-based disclosure of substance use could be easier than disclosing in person due to reduced stigma. However, participants expressed concerns about not knowing who would have access to this information.

**Conclusions:**

Incorporating substance use supports into a pregnancy app was found to be acceptable among those using substances. Participants reported frequent baseline use of prenatal apps, showed a high level of engagement with the pregnancy app during the study, and demonstrated interest in expanding the substance use support elements of this app. Embedding substance use screening, information, and connection to care into a tool with wide-scale use during pregnancy has the potential to identify at-risk individuals who may otherwise not be identified during routine prenatal care. It also has the potential to connect individuals, who might otherwise be hesitant to disclose their substance use, to recovery or harm reduction resources.

## Introduction

Substance use during pregnancy has increased significantly over the past two decades [1-4], with almost 3% of pregnant people having a formal substance use disorder diagnosis [5]. Opioid use disorder during pregnancy has more than quadrupled in the past 20 years and is now a leading cause of maternal death in the United States [6-12]. Observed rates of other substance use during pregnancy have also increased in recent years, including cannabis, alcohol, sedatives, and stimulants. Actual numbers may be higher due to lack of reporting [5]. Additionally, concurrent substance use, such as marijuana use paired with opioid use, can further increase the risk of preterm birth and low birth weight infants [13] and may impact infant development and longer-term learning, memory, and impulsivity [10,11]. This increase in the prevalence of substance use is concerning and suggests an increased need for effective screening and resource provision to pregnant people.

Early identification and intervention are critical for reducing adverse maternal and neonatal outcomes associated with substance use [14]. As a result, major professional organizations, including the American College of Obstetricians and Gynecologists, recommend that all pregnant people be verbally screened for substance use with a validated tool at least once during pregnancy [15,16]. Despite this, 20%‐30% of obstetric providers do not routinely screen for substance use, and less than half routinely refer patients with a positive screen to substance use treatment resources [17,18]. Moreover, due to stigma, judgment, and fear of mandated reporting requirements, many pregnant people are hesitant or choose not to disclose substance use to their health care providers [12,19-21]. In a study of 422 pregnant people who presented for their first obstetric appointment, 46% of those who had a urine drug test positive for a nonprescribed substance chose to not disclose their substance use when verbally screened by their provider [22].

Mobile health (mHealth) technology has been successfully used to evaluate and deliver interventions related to tobacco, alcohol, and illicit substances, including opioid use, in nonpregnant populations [23-25]. In a study evaluating interest in using digital platforms to monitor recovery trajectories among 259 patients in substance use treatment, 70% of participants expressed interest in using a relapse prevention app [26]. Additionally, a study of 202 individuals using a recovery support app demonstrated high levels of usage of the app and articulated that further expanding the app to provide additional recovery-related resources could increase the likelihood of continuing to use the app in the future [27]. Further, in an evaluation of 316 patients engaged in a Veteran’s Affairs (VA) substance use treatment program, more substance use was disclosed through an indirect method (self-completed questionnaire), as opposed to a direct method (verbal disclosure), suggesting that creating an accessible space for indirect disclosure may be beneficial for people who use substances [28].

Pregnancy apps are a common information source used by patients during pregnancy and allow for intimate, self-guided touchpoints for those seeking health-related resources, guidance, and information outside of clinical care settings [29,30]. As such, the American College of Obstetricians and Gynecologists supports mHealth as a suitable means for supplementing obstetric health care [31]. Due to their frequent use, mHealth tools may be an additional way to identify patients who are unwilling to disclose their substance use in traditional, in-person health care settings by mitigating stigma and bias associated with in-person substance use evaluations [29,32].

The purpose of this study was to explore the usability and acceptability of an existing prenatal mHealth app, MyHealthyPregnancy, as a tool in which substance use screening, education, and information could be provided as part of routine prenatal care among pregnant people with substance use. For an mHealth app to be acceptable, the patient population of interest must generally engage with mobile technology during their pregnancy, demonstrate interest in accessing information from the app, and importantly, trust the app as a setting for substance use disclosure [33-36]. Therefore, the specific objectives of this study were to understand, among pregnant people with a history of opioid or other addictive substance use, (1) general technology and mobile app usage, (2) willingness to disclose substance use through a pregnancy app, (3) interest in obtaining substance use education and information through a prenatal app, and (4) perspectives regarding how a pregnancy app could assist with substance use–related needs.

## Methods

### Study Sample

From April to August 2021, we conducted a mixed methods, exploratory pilot study to understand the usability and acceptability of a prenatal support app for substance use screening, education, and information during pregnancy. Pregnant people with substance use were recruited from inpatient and outpatient settings at an academic, tertiary care women’s hospital including prenatal clinics, substance use treatment programs, and inpatient antepartum hospital units. Participants were eligible if they were pregnant, at least 18 years of age, less than 37 weeks gestation, had regular access to a smartphone (Android or iOS), and had a history of substance use during or within 3 months prior to their pregnancy, as determined through either self-report, *ICD-10* (*International Classification of Diseases*, *Tenth Revision*) diagnoses coding cord or evidence of substance use on urine drug testing in the electronic health record. In prior research, a sample size of approximately 30 participants has been determined to be sufficient to understand app usability and acceptability [37].

### Ethical Considerations

The study was reviewed by the University of Pittsburgh institutional review board (STUDY18120026). Participants provided written informed consent. All audio and transcribed materials were stored on a secure, password- and firewall-protected university network drive or server, and all data were deidentified prior to analysis. Participants received US $25 for their participation in this study.

### MyHealthyPregnancy mHealth App

The MyHealthyPregnancy app is a pregnancy mHealth tool that offers evidence-based, prenatal educational content organized by the user’s weeks of gestation, a diary to document the user’s pregnancy experiences, a fetal movement counter, a contraction timer, and routine screenings for symptoms and psychosocial risks. The app also functions as a risk assessment tool that may use patient-entered data (eg, symptoms, language, mood, sleep, and psychosocial screeners) to identify possible risks during pregnancy (eg, intimate partner violence, depression, and pre-eclampsia) and resources tailored to the risks identified. When users start the onboarding process to begin using the app, they are asked, “Do you currently use any of the following?” for various substances, including alcohol, tobacco (and vaping), marijuana, narcotics or opioids, heroin or fentanyl, benzodiazepines, cocaine or amphetamines, and other drugs. Depending on the substance disclosed, the user will be directed to substance-specific local, regional, and national recovery resources.

Educational content about substance use (eg, content regarding the risks of substance use during pregnancy, information on substance use treatment resources) is accessible to users through the app’s “Learning Center” and “Resources” sections. These resources and articles were designed, in collaboration with clinical experts, to offer the same information as would be provided in routine prenatal care if substance use was disclosed to a provider. MyHealthyPregnancy was launched for beta-test evaluation at the University of Pittsburgh Medical Center health system in September 2019, with research demonstrating its effectiveness as a complementary tool to prenatal care [38-42].

### Study Procedures

**Figure 1.**
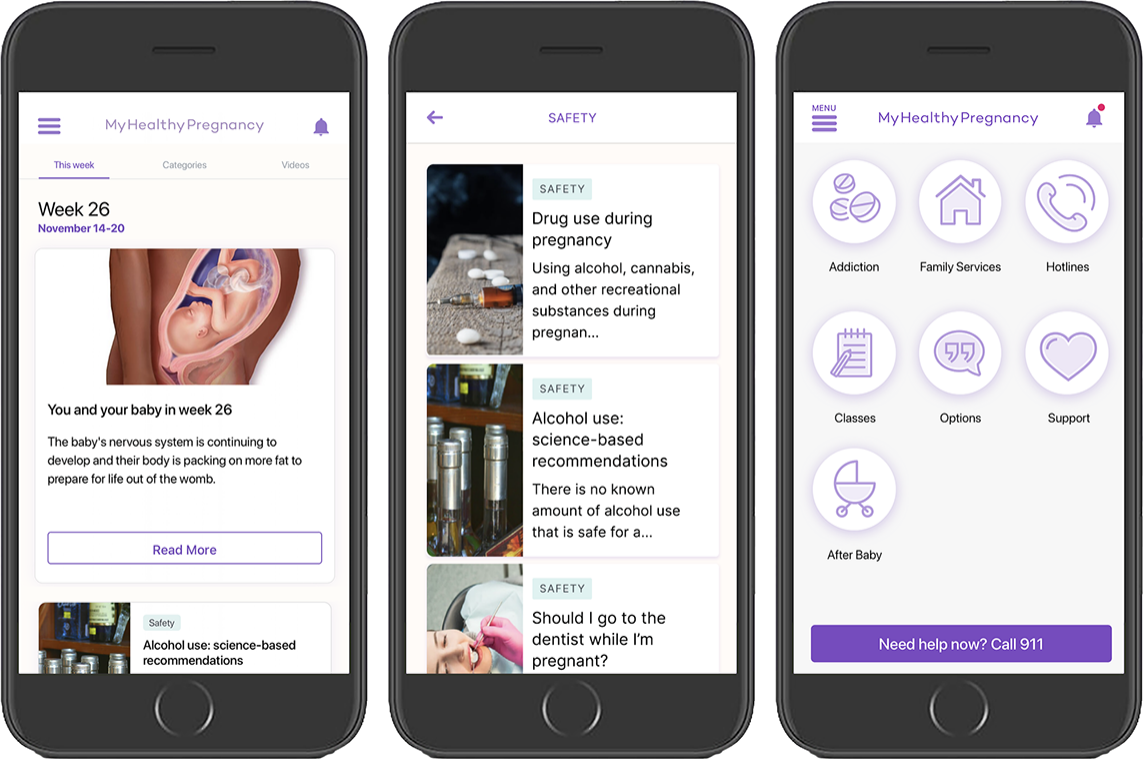
MyHealthyPregnancy sample screens offering substance use–related content and resources.

After signing a written informed consent, participants completed a baseline survey assessing demographics, substance use, pregnancy history, and technology and app use behaviors (Multimedia Appendix 1). Research staff then assisted participants with downloading the app on their smartphones and provided an overview of the sections of the MyHealthyPregnancy app before having participants self-navigate through the app on their phones. At this time, participants additionally consented to the sharing of identifiable data for research purposes and the publication of anonymized aggregate data through the app. Figure 1 shows sample screens from the app.

After navigating through each section of the app, research staff asked participants to relay their thoughts regarding the app and substance use–related content through a “think-aloud” technique [43-46]. Following the think-aloud sessions, participants engaged in a cognitive interview to further understand the acceptability of the app for substance screening and evaluation [47-51]. Examples of questions used during the cognitive interview included, “If you were actively using a substance, how do you think that you may feel about disclosing substance use on an app?”, “How do you feel about tracking substance use–related information in an app?”, and “Is there information you feel more comfortable sharing through the app compared to when you are talking directly to your provider?” Participants also completed a usability survey regarding the app’s features (Multimedia Appendix 2) [52,53]. Participants were then encouraged to use the app as much or as little as they wished over the next 4 weeks.

At-home app usage was measured and transmitted through a secure HIPAA (Health Insurance Portability and Accountability Act)-compliant server to the research staff. For privacy protection, access to the app was protected by a password set up by the individual user. The password reset function required app users to provide their unique user ID and then complete instructions on resetting their password via a personalized SMS text message sent to their own, prespecified contact number. For sensitive questions embedded in the app (eg, substance use, reports of intimate partner violence), an icon was displayed that reminded users that information they shared might be transmitted to their health care provider. Participants were then recontacted by research staff by phone or in person to complete a brief follow-up survey regarding their impressions of independently using the app and any additional feedback or suggestions (Multimedia Appendix 3). The think-aloud sessions, cognitive interviews, and follow-up interviews were audio-recorded.

### Data Analyses

#### Quantitative Analyses of Usability and Acceptability

Preliminary analyses evaluated survey and app usage data for completeness and accuracy and addressed any issues with data quality. Summary statistics were used to describe the participant characteristics, technology and mobile app use behaviors, assessments of app usability and acceptability, and participant-endorsed options for how a pregnancy app could assist with substance use. These statistics include means and standard deviations (or medians and quartiles for skewed distributions) for continuous variables and frequencies and proportions for categorical variables. Where appropriate, 95% CIs are included for means and proportions. IBM SPSS Statistics for Windows version 26 was used for quantitative statistical analysis [54].

#### Qualitative Analyses of Usability and Acceptability

Audio recordings from think-aloud sessions, cognitive interviews, and follow-up interviews were transcribed verbatim. Qualitative analyses were conducted using the Consolidated Framework for Implementation Research (CFIR)-based deductive analysis approach (directed content analysis), which is a rapid qualitative analytic method [53]. Prior to conducting the interviews, a preliminary codebook (in Microsoft Excel, version 16.54) was created by the research team. After the interviews were conducted, HF reviewed the transcripts using notes taken during the interviews to identify any additional codes or themes focused on understanding participants’ experiences and perceptions of the app. The revised codebook was then used to code the transcripts for perspectives regarding the incorporation of substance use information in a pregnancy mHealth app.

## Results

### Study Sample

Over a 4-month enrollment period, 66 pregnant individuals were screened for eligibility, 24 declined to participate, and 6 did not meet study eligibility criteria. Among those that declined, the reasons provided were lack of interest in the study and limited time to attend study debriefs. Among those who did not meet the eligibility criteria, 5 participants did not have access to a smartphone due to rehabilitation facility or sober community living rules, and 1 participant was more than 37 weeks pregnant. Among the 36 participants who enrolled, 3 participants chose not to continue the study prior to using the app, 3 participants were unable to be reached for follow-up assessments, and 2 participants were found to no longer meet eligibility criteria due to fetal demise or lack of consistent access to a smartphone. Thus, 28 participants completed all study tasks and created the analytic sample.

Table 1 describes the characteristics of study participants. Most participants were White (21/28, 75%), Medicaid insured (26/28, 93%), and had an annual household income of less than US $30,000 (16/28, 57%). For 18% (5/28) of participants, this was their first pregnancy. The mean (SD) gestational age of participants at the time of enrollment was 22 (7.3) weeks. Substances used among participants consisted of tobacco (22/28, 79%), marijuana (20/28, 71%), illicit opioids (9/28, 32%), alcohol (6/28, 21%), stimulants (4/28, 14%), including either cocaine or amphetamines, and benzodiazepines (2/28, 7%). Approximately 46% (13/28) of participants were currently taking medication for opioid use disorder (eg, methadone or buprenorphine). Some individuals (4/28, 14%) used marijuana only. All remaining participants used a combination of 2 or more substances.

**Table 1.** Participant characteristics (n=28).

Demographics			Values
Age (years), mean (SD)			31 (4.6)
Race, n (%)			
	White		21 (75)
	Multiracial		5 (18)
	Black or African American		2 (7)
Ethnicity, n (%)			
	Hispanic or Latino		2 (7)
Marital status, n (%)			
	Married		7 (25)
Insurance, n (%)			
	Medicaid		26 (93)
Highest level of completed education, n (%)			
	Some high school		7 (25)
	High school or general equivalency diploma		8 (29)
	Some college, trade school, or associate’s degree		11 (39)
	Bachelor’s degree		1 (4)
	Master’s degree		1 (4)
Employment, n (%)			
	Full- or part-time employed		9 (32)
Income (US $), n (%)			
	<30,000		16 (57)
	30,000‐60,000		3 (11)
	60,000 or more		4 (14)
	Unsure		4 (14)
Pregnancy history			
	Gestational age at enrollment (weeks), mean (SD)		22 (7.3)
	Primiparous, n (%)		5 (18)
Substance use history,^a^ n (%)			
	Tobacco		22 (79)
	Marijuana		20 (71)
	Opioids		15 (54)
		MOUD^b^	13 (46)
		Illicit opioid use	9 (32)
	Alcohol		6 (21)
	Stimulants (ie, cocaine and amphetamines)		4 (14)
	Benzodiazepines		2 (7)

a Type of substances used within 3 months prior to pregnancy or during pregnancy.

b MOUD: medication for opioid use disorder.

Table 2 describes the technology behaviors and mobile app usage of participants. Texting (27/28, 96%) was the most commonly reported mode of communication, followed by phone (22/28, 79%), email (18/28, 64%), and social media (16/28, 57%). Health care providers (20/28, 71%) and mHealth apps (3/28, 11%) were the most frequently reported sources of pregnancy-related information followed by the internet or websites, family members, and friends. Health care providers were also noted to be the most trusted source of pregnancy-related information (27/28, 96%). mHealth apps were commonly used by participants, with 68% (19/28) reporting the use of a pregnancy app at the time of their enrollment and 26% (5/19) reporting that they used 2 or more pregnancy apps.

**Table 2.** Technology and mobile app use behaviors (n=28).

Technology use and communication during pregnancy		Values, n (%)
Source *most frequently* used for pregnancy information		
	Health care provider	20 (71)
	mHealth^a^ apps	3 (11)
	Internet or websites	2 (7)
	Family members	2 (7)
	Friends	1 (4)
Source *most trusted* for accurate pregnancy information		
	Health care provider	27 (96)
	mHealth apps	1 (4)
Preferred methods of communication		
	Texting	27 (96)
	Phone	22 (79)
	Email	18 (64)
	Social media platforms	16 (57)
	Video calling or conferencing	11 (39)
	Apps	2 (7)
Smartphone ownership		28 (100)
Pregnancy app use (yes/no)		19 (68)
	Use of 2 or more pregnancy apps	5 (26)

a mHealth: mobile health.

### Quantitative Analyses of Usability and Acceptability

Table 3 describes participants’ experiences with the app. Measurements of the *usability* of the MyHealthyPregnancy app include participants’ level of satisfaction with the ease of using the app and satisfaction with the time it took to use the app. Measurements of the app’s *acceptability* include participants’ overall satisfaction with the app, satisfaction with the interface, and how helpful the app was as a source of support. During the 4-week period, daily app use was the most common (12/28, 43%), and educational materials related to substance use were engaged with by the majority of participants (19/28, 68%). Acceptability and usability were generally high.

**Table 3.** MyHealthyPregnancy usability and acceptability (n=28).

	Values, n (%)
Acceptability	
The app was a helpful source of support in pregnancy	27 (96)
Liked the way the app looks	27 (96)
Overall, I am satisfied with the interface of the app	25 (89)
Overall, I am satisfied with the app in general	25 (89)
Usability	
I am satisfied with the ease of using the app	23 (82)
I am satisfied with the time it took to use the app	22 (79)
It was easy to navigate through the app	27 (96)
Engagement^a^	
Daily	12 (43)
Weekly	8 (29)
Monthly	5 (18)
Substance use resource utilization	
Participants who browsed substance use educational materials	19 (68)
Participants who accessed substance use treatment referral information	2 (7)

a Frequency of app logins within a 4-week period.

Participant-endorsed options for how a pregnancy app could assist with substance use–related behaviors are described in Table 4. Almost all participants (27/28, 96%) expressed a desire to use an app to block phone calls or SMS text messaging from people who had a negative effect on substance use behavior, while more than half desired the ability to track substance use, cravings, or treatment medications. The least endorsed options included information on infectious disease prevention (5/28, 18%), harm reduction (4/28, 14%), or intimate partner violence (3/28, 11%).

**Table 4.** Participant endorsed options for how a pregnancy app could assist with substance use (n=28).

Options	Values, n (%)
Blocking phone calls or SMS text messaging from people who have had a negative effect on recovery	27 (96)
Tracking incidences of substance use or cravings	19 (68)
Receiving reminders to take substance use treatment medications (eg, MOUD)^a^	16 (57)
Information about support groups for parents with substance use disorders	12 (43)
Neonatal opioid withdrawal syndrome information	9 (32)
Infectious disease prevention information	5 (18)
Harm reduction information	4 (14)
Intimate partner violence resources	3 (11)

a MOUD: medication for opioid use disorder (eg, methadone or buprenorphine)

### Qualitative Analyses of Usability and Acceptability

In cognitive interviews, participants shared their perspectives regarding substance use disclosure through a pregnancy app and how a pregnancy app could be useful for people using substances or with a substance use disorder. Five major themes were identified from these debriefs (Table 5). Participants felt that substance use disclosure on an app may be associated with less stigma than in-person disclosure (theme 1). However, they also noted that their comfort with disclosure would vary by the type and legality of the substance (theme 2). Participants expressed concerns related to who could access substance use information (eg, health care professionals and social services providers) on a pregnancy app and concerns related to being permanently labeled as someone with an addiction (theme 3). Despite concerns, participants did believe that pregnancy apps could be a useful source of substance use information and education (theme 4) and felt that combining pregnancy and substance use information on a single app was the most desirable approach (theme 5).

**Table 5.** Participant perspectives regarding disclosure and incorporation of substance use information in a pregnancy mobile health app.

Theme	Example quote
Theme 1: Substance use disclosure on an app may be associated with less stigma than in-person disclosure	“I would feel a little more comfortable if I put it in the app than talking to an individual. The app doesn’t judge you, like a human being would”
Theme 2: Disclosure comfort varies by type and legality of substance	“I definitely felt more comfortable disclosing legal drug use. For example, I do have my medical marijuana card, that is something I have no problem sharing, or alcohol and cigarettes, but yes, the non-legal ones, I would be a little nervous.”
Theme 3: Concerns related to who could access disclosed substance use information	“Depending on what information I divulged, what would happen to that information? For example, would that be sent to a doctor? If it would be sent to a doctor or another professional or even a social worker… even though it would be confidential, I would just be worried I’d be labeled...and I will never be able to get away from my past addiction.”
Theme 4: Prenatal apps could be a useful source of substance use information, education, and resources	“For the women who have no support or anyone to talk to, I really do think it is an effective tool to give them a little bit of encouragement and accurate information or just a guide: here are some resources, here is someone you could talk to.”
Theme 5: Combining pregnancy and substance use information on a single app is desired	“Having it within a pregnancy app is kind of better personally just because it is all in one place...I’ve had recovery apps before, and I have fallen off of them. Actually, I completely forgot about them. So, like with pregnancy and having a kid and like getting ready for stuff, the simpler the better. So, if I don’t have to go to multiple apps, that is great.”

## Discussion

### Principal Results

Opioid use and the use of other addictive or illicit substances remain a significant and growing public health crisis in the United States. One significant barrier to providing early intervention during pregnancy is identifying individuals at risk and connecting them to care in a way that feels comfortable to them and through a mechanism which is engaging. One solution to identifying and engaging with pregnant individuals using opioids and other substances is through prenatal care apps. However, reaching pregnant individuals who use substances through more holistic prenatal care apps requires that the prenatal app be engaging and that app users who use substances must be willing to share their substance use through the app and act upon the information provided to them about recovery.

In this mixed methods, exploratory pilot study, incorporating substance use screening, information, education, and support into an existing prenatal app was found to be acceptable among pregnant people with substance use. Overall, participants showed high levels of interest in and engagement with MyHealthyPregnancy. Moreover, they reported being generally supportive of using an mHealth app as a means to access nonjudgmental resources for substance use during pregnancy. Our findings also indicate that there are multiple ways that an app could support individuals who use substances including blocking phone calls or SMS text messaging, providing information about recovery-oriented support groups, and offering resources regarding common co-occurring conditions (infectious disease acquisition, intimate partner violence, and harm reduction) [55].

Consistent with prior research demonstrating a high level of mHealth app usage among pregnant people (greater than 50%), most participants (19/28, 68%) reported that they had already used a prenatal app before starting the study, with some participants reporting the use of multiple prenatal apps [30,56-58]. In our study, apps were also identified, more generally, as a trustworthy, and easy-to-access source of pregnancy-related information, second only to health care providers. This aligns with other research findings demonstrating pregnant patients’ appreciation for the accessibility and reliability of information found in prenatal apps [59]. Our study demonstrated a high level of engagement with our prenatal app, with 71% (20/28) logging in on a daily or weekly basis, similar to engagement levels that are considered high among other prenatal apps[60]. Together these findings suggest that a prenatal app-based intervention could be a beneficial strategy for information sharing between pregnant people who use substances and their providers, as this population already engages with and trusts this type of technology.

Our findings also indicate a high level of acceptability with incorporating substance use–related information into a pregnancy app. App usage data indicated that many participants browsed recovery-related educational materials on the app. These data align with other research demonstrating that people generally do not like moving between different apps to achieve their goals and prefer integrated technology tools, as well as prior findings showing that substance use screening and intervention in an app-based format has high acceptability and usability among patients [61,62]. Our participants expressed their interest in an expansion of substance use support capacity of the prenatal app, in alignment with prior research demonstrating that mHealth interventions are effective in areas including smoking cessation and addressing substance use during pregnancy[63]. Finally, many participants volunteered that disclosing substance use on an app may be easier than disclosing substance use in person, aligning with the current literature suggesting that self-report questionnaires and eHealth screenings could assist in creating more opportunities for disclosure than in-person evaluations alone [28,29,64].

However, many participants expressed concerns about disclosing substance use on an app because they would not know who might have access to this information, similar to prior research demonstrating patient concern about data security even in general prenatal apps [58]. Concerns about prosecution and child welfare involvement are previously reported barriers to substance use disclosure, which can lead to delays in seeking prenatal care and engaging in substance use treatment [65,66]. Mandatory reporting laws and the potential for child protective services involvement are major barriers to seeking treatment among pregnant people with opioid use disorder [67]. Any app that collects sensitive patient health information is required to comply with HIPAA. Moreover, any app that offers a substance use disorder treatment service must comply with the Opioid Addiction Recovery Fraud Prevention Act of 2018, which requires transparent and fair practices around how private health information is shared [68]. However, concerns about seeking and engaging in substance use treatment during pregnancy legitimately extend to disclosure and substance use treatment seeking through digital health means. In prior qualitative research, parents note that mandatory reporting regulations are biased, unjust, and stigmatizing and assert that stress stemming from the potential involvement of child welfare agencies has had a pronounced and detrimental impact on their families [67,69,70]. Moreover, since the 2022 *Dobbs v. Jackson* Supreme Court decision that ended federal protections for abortion, there have been increasing reports of digital health data being subpoenaed to criminalize pregnant individuals, further legitimizing caution around disclosure of certain health behaviors, especially during pregnancy [71,72].

Given both the concerns and interest voiced by our participants regarding embedding substance use screening and connection to care into a prenatal app, providers should familiarize themselves with any prenatal or substance use support apps available to patients prior to recommending them, including evaluating the evidence base, equity focus, and HIPAA protections afforded to such tools [73]. We additionally suggest that any providers discussing such apps with patients also educate patients about their rights and privacy related to disclosing substance use or other sensitive information in these apps. Providers can also encourage patients to share feedback about which apps they have found useful (or not) to support provider recommendations.

### Limitations

There were several study limitations. First, approximately 36% (24/66) of those approached for study participation declined to enroll. Because many of those who declined to participate lacked interest in study participation, our sample may have been biased toward those who are more willing to use mHealth technology for health engagement or toward those with fewer reservations about disclosing substance use. In addition, all participants enrolled in the study had a known history of substance use. As such, their behaviors and perspectives may not be generalizable to pregnant people who have never disclosed their substance use within a health care setting. Next, our study sample was small and consisted of predominantly non-Hispanic White individuals, which limits the degree to which we can generalize our findings. Opioid overdose rates among Black individuals exceed those of White individuals by 4-6 times, and there are significant disparities in the receipt and use of medication for opioid use disorder between non-Hispanic White and Black and Hispanic pregnant individuals [74,75]. Thus, our findings cannot be generalized to those who may be at highest risk of not receiving adequate care. While the sample size for this exploratory pilot study was aligned with norms for thematic saturation for qualitative interview feedback to offer initial data on acceptability, a larger randomized trial with intentional demographic sampling would be required to adequately test implementation or intervention effects of incorporating substance use screening and content into the mHealth tool [76-78].

### Conclusions

In this study, pregnant people with substance use found an existing pregnancy app to be an acceptable means of incorporating substance use supports and screening. Participants were already frequent users of prenatal apps and showed a high level of engagement with the prenatal support app in the study. At the completion of the study, participants expressed positive feelings about the usability of the app and interest in expanding the of the substance use support features of this app although some notable concerns relating to data privacy were raised. Given the prevalence of technology and app usage among pregnant people and the rise in substance use during pregnancy, mHealth technology should be considered a complement to in-person prenatal substance use screening and evaluation. Providing opportunities for substance use disclosure and resource provision through an acceptable digital platform that people are already using for pregnancy could result in earlier treatment engagement, along with improved outcomes among those who use substances. Our findings highlight this as an acceptable and desirable approach.

There is an ethical imperative for any prenatal app incorporating substance use–related content to clearly communicate both the confidentiality constraints of the tool and the potential consequences of disclosure prior to screening. As a way to address app users’ concerns, developers should consider designing data collection so that any sensitive data are deleted as soon as possible or are only stored locally and not in a location that could be at risk of subpoena. App developers should also be sure to communicate clearly to app users about privacy and seek active consent for the collection or storage of any sensitive data. Finally, app developers should consider instating a policy prohibiting engagement in third-party data sharing, with the exception of HIPAA-compliant data sharing with the health care provider. Policy makers should ensure clear communication with providers about their rights related to substance use disclosure during pregnancy and potential legal repercussions. Health care providers considering using pregnancy apps, particularly for substance use screening or treatment referral, should evaluate whether the apps are evidence-based and adherent to strict policies around data protections. This may include being proactive about self-education regarding HIPAA privacy rules, as well as the state and institutional protections in place, so that they can communicate these clearly to their patients when recommending that patients share sensitive information through these tools.

Lastly, additional research is needed to prospectively evaluate and test patient-centered substance use screening and connection to recovery resources within pregnancy apps*,* as well as to understand and measure the rates of disclosure rates across substances. We recommend that any individuals involved in the development, use, or evaluation of pregnancy tools, which may provide these services, draw on established frameworks that center the needs and values of the target population, while proactively addressing health disparities as a strategy to advance health equity and improve health outcomes [73]. Through a personalized approach to health care, prenatal health apps can play a role in identifying and supporting pregnant people with substance use.

## Supplementary material

10.2196/60038 Multimedia Appendix 1 Baseline survey questions.

10.2196/60038 Multimedia Appendix 2 Usability survey questions.

10.2196/60038 Multimedia Appendix 3 Follow-up survey questions.

## Abbreviations

CFIR: Consolidated Framework for Implementation Research
HIPAA: Health Insurance Portability and Accountability Act
*ICD-10*: *International Classification of Diseases, Tenth Revision*
mHealth: mobile health
VA: Veterans Affairs
