# Supplementary material for: Usability and Acceptability of a Pregnancy App for Substance Use Screening and Education: A Mixed Methods Exploratory Pilot Study
Source: JMIR Pediatr Parent. 2025 Feb 13;8:e60038. doi: 10.2196/60038 (PMC11841748; doi:10.2196/60038)
Supplement: Multimedia Appendix 1 [file pediatrics-v8-e60038-s001.docx]

**Baseline Survey Questions**

| How many weeks pregnant are you? |
| --- |
| Counting this pregnancy, how many times have you been pregnant in your lifetime? (including any abortions or miscarriages) |
| Where do you get information about your pregnancy? (select all that apply)   1. Healthcare provider (i.e. doctor, midwife, nurse, nurse practitioner) 2. Family members 3. Friends 4. Social media (i.e. Facebook, Twitter, Instagram) 5. Internet websites 6. Smartphone apps 7. Other |
| Which information source do you use **MOST FREQUENTLY** to get information about your pregnancy?   1. Healthcare provider (i.e. doctor, midwife, nurse, nurse practitioner) 2. Family members 3. Friends 4. Social media (i.e. Facebook, Twitter, Instagram) 5. Internet websites 6. Smartphone apps 7. Other |
| Which information source do you **TRUST** the most to get accurate information about your pregnancy?   1. Healthcare provider (i.e. doctor, midwife, nurse, nurse practitioner) 2. Family members 3. Friends 4. Social media (i.e. Facebook, Twitter, Instagram) 5. Internet websites 6. Smartphone apps 7. Other |
| Which of the following methods of communication do you use? (select all that apply)   1. Phone 2. Text 3. Communication apps (i.e. WhatsApp, TextMe, TextNow, Magiciak, Voxer) 4. Video calling (i.e. Skype, Facetime) 5. Social media (i.e. Facebook, Twitter, Instagram, Snapchat) 6. Email |
| What type of phone do you currently use?   1. Personal smartphone 2. Shared smartphone (i.e. with partner, friend, family member) 3. Cell phone without internet capabilities 4. Government phone ("Obama Phone") 5. Trac Fone (i.e. prepaid cellphone, minute cellphone) 6. Landline 7. I do not have consistent access to a phone |
| What type of smartphone device do you have?   1. iPhone 2. Android 3. Other |
| Do you currently use any smartphone apps to track your pregnancy?   1. Yes 2. No |
| If yes, please list the name(s) of the app(s). |
| Would any of the following additional information be helpful to track on a smartphone app to aid in your substance use and/or recovery? (select all that apply)   1. Medication tracking/reminders 2. Infectious disease (i.e. HIV, Hepatitis C) prevention information 3. Information about support groups for moms who have substance use disorders 4. Intimate partner violence resources 5. Neonatal opioid withdrawal syndrome (i.e. NOWS/NAS) information 6. Harm reduction (i.e. clean needle usage) information 7. Other |
| If you could block or prevent phone calls or texts from people that have a negative effect on you and/or your recovery, would you do so?   1. Yes 2. No |
| If yes, how helpful would it be to block or prevent phone calls or texts from people that have a negative effect on you and/or your recovery?   1. Not helpful at all 2. Slightly helpful 3. Moderately helpful 4. Very helpful 5. Extremely helpful |
| How old are you? |
| How would you describe your race?   1. American Indian/Alaska Native 2. Asian 3. Native Hawaiian or Other Pacific Islander 4. Black or African American 5. White 6. Multiracial 7. Other |
| How would you describe your ethnicity?   1. Hispanic or Latino 2. Non-Hispanic or Latino |
| What is your marital status?   1. Single (current partner) 2. Single (no partner) 3. Married |
| What kind of medical insurance do you have?   1. Medical Assistance (Medicaid, UPMC for You, Gateway) 2. Private Plan (UPMC HMO/PPO, Highmark, Aetna) 3. In the process of applying 4. None 5. Other |
| What is the highest level of education you have completed?   1. Some high school 2. High school/GED 3. Trade/Technical/Vocational training/associate’s degree 4. Some college credit, no degree 5. College/bachelor’s degree 6. Master's degree |
| What is your employment status?   1. Employed full time (35+ hours per week) 2. Employed part time 3. Unemployed, looking for work 4. Unemployed, not looking for work 5. Unemployed, student 6. Other |
| What is your annual household income?   1. $0-$9,999 2. $10,000-$19,999 3. $20.000-$29.999 4. $30,000-$39,999 5. $40,000-$49,999 6. $50,000-$59,999 7. $60,000-$69,999 8. $70,000+ 9. Unsure |
