# Supplementary material for: Usability and Acceptability of a Pregnancy App for Substance Use Screening and Education: A Mixed Methods Exploratory Pilot Study
Source: JMIR Pediatr Parent. 2025 Feb 13;8:e60038. doi: 10.2196/60038 (PMC11841748; doi:10.2196/60038)
Supplement: Multimedia Appendix 2 [file pediatrics-v8-e60038-s002.docx]

**Usability Survey Questions**

| Overall, I am satisfied with the **amount of time** it took to complete the first set of questions in the app   1. Strongly disagree 2. Disagree 3. Neutral 4. Agree 5. Strongly agree |
| --- |
| Overall, I am satisfied with the **amount of time** it took to complete the check-in questions.   1. Strongly disagree 2. Disagree 3. Neutral 4. Agree 5. Strongly agree |
| Overall, I am satisfied with the **ease of navigation** when using the app.   1. Strongly disagree 2. Disagree 3. Neutral 4. Agree 5. Strongly agree |
| Overall, I am satisfied with the **interface (i.e. design of main dashboard, placement of buttons, ability to interact)** of this app.   1. Strongly disagree 2. Disagree 3. Neutral 4. Agree 5. Strongly agree |
| Overall, I am satisfied with this app **in general.**   1. Strongly disagree 2. Disagree 3. Neutral 4. Agree 5. Strongly agree |
| Is it easy for you to navigate through the app?   1. Yes 2. No |
| Do you like the way the MyHealthyPregnancy app looks?   1. Yes 2. No |
