# Supplementary material for: Usability and Acceptability of a Pregnancy App for Substance Use Screening and Education: A Mixed Methods Exploratory Pilot Study
Source: JMIR Pediatr Parent. 2025 Feb 13;8:e60038. doi: 10.2196/60038 (PMC11841748; doi:10.2196/60038)
Supplement: Multimedia Appendix 3 [file pediatrics-v8-e60038-s003.docx]

**Follow-Up Survey Questions**

| How helpful has this app been as a source of support in your pregnancy?   1. Not helpful at all 2. Slightly helpful 3. Moderately helpful 4. Very helpful 5. Extremely helpful |
| --- |
| If you could keep track of or record how often you have cravings or feel the desire to use substances in the MyHealthyPregnancy app, would you do so?   1. Yes 2. No |
| If yes, how helpful would it be to track how often you have cravings or feel the desire to use substances in the MyHealthyPregnancy app?   1. Not helpful at all 2. Slightly helpful 3. Moderately helpful 4. Very helpful 5. Extremely helpful |
